# Neutralizing antibodies in milk and blood of lactating women vaccinated for SARS-CoV-2: a systematic review

**DOI:** 10.1590/1984-0462/2025/43/2023210

**Published:** 2024-09-06

**Authors:** Ianne Stéfani Angelim Vieira, Fernanda Mazzoli da Rocha, Marina Vilarim, Fernanda Rebelo, Daniele Marano

**Affiliations:** a Fundação Oswaldo Cruz, Instituto Nacional da Saúde da Mulher, da Criança e do Adolescente Fernandes Figueira, Rio de Janeiro, RJ, Brazil.

**Keywords:** COVID-19, Human milk, Vaccine, Antibodies, Systematic review, COVID-19, Leite humano, Vacina, Anticorpos, Revisão sistemática

## Abstract

**Objective:**

To compare the presence of neutralizing antibodies against SARS-CoV-2 found in the breast milk and blood of vaccinated lactating women with those not vaccinated.

**Data source:**

The study was registered in the International Prospective Register of Systematic Reviews (PROSPERO) under CRD42021287554 and followed the Preferred Reporting Items for Systematic Reviews and Meta-Analyses (PRISMA) guidelines. Cohort, case-control, and cross-sectional studies that evaluated antibodies against SARS-CoV-2 in the milk and blood of vaccinated mothers and had as control group unvaccinated mothers were eligible. Health Sciences Descriptors (DeCs), Medical Subject Headings (MeSH) and Emtree descriptors were used for the Virtual Health Library (VHL), Medical Literature Analysis and Retrieval System Online (Medline/Pubmed), and Embase databases, respectively. In the Web of Science and Scopus, the strategy was adapted. No restrictions on the publication period and language were set.

**Data synthesis:**

The search identified 233 records, of which 128 duplicates and 101 papers that did not meet the inclusion criteria were excluded. Hence, four cohort studies were eligible. Nursing mothers vaccinated with the Pfizer-BioNTech and Moderna vaccines showed antibodies against SARS-CoV-2 in their blood and breast milk.

**Conclusions:**

Vaccinated lactating women had higher levels of immunoglobulin G (IgG) and A (IgA) in serum and breast milk than unvaccinated women.

## RESUMO

**Objetivo:**

Comparar a presença de anticorpos neutralizantes contra o SARS-CoV-2 no leite materno e no sangue das lactantes vacinadas em relação àquelas não vacinadas.

**Fontes de dados:**

Foi efetuado registro no International Prospective Register of Systematic Reviews — PROSPERO (CRD42021287554) e foram seguidas as diretrizes do Preferred Reporting Items for Systematic Reviews and Meta-Analysis (PRISMA). Foram elegíveis estudos de coorte, caso-controle e transversais que avaliaram anticorpos contra o SARS-CoV-2 no leite e no sangue de lactantes vacinadas e tiveram como grupo controle lactantes não vacinadas. Utilizaram-se os Descritores em Ciências da Saúde (DeCs), Medical Subject Heading (MeSH) e Emtree para as bases Biblioteca Virtual em Saúde (BVS), Medical Literature Analysis and Retrieval System Online (Medline/PubMed) e Embase, respectivamente. Na Web of Science e Scopus foi feita adaptação da estratégia. Não foram estabelecid as restrições quanto ao período de publicação e idioma.

**Síntese dos dados:**

As buscas identificaram 233 registros. Foram excluídos 128 duplicados e 101 fora dos critérios de inclusão, e quatro estudos de coorte foram elegíveis. As lactantes vacinadas com Pfizer-BioNTech e Moderna apresentaram anticorpos contra SARS-CoV-2 no sangue e no leite materno.

**Conclusões:**

As mulheres lactantes vacinadas apresentaram níveis mais elevados de imunoglobulina G (IgG) e A (IgA) no soro e no leite materno em comparação com as mulheres não vacinadas.

## INTRODUCTION

The unbridled spread of COVID-19 cases mobilized scientists around the world to develop vaccines with different technologies to combat the virus. The first immunizers available in the United States (Pfizer-BioNTech and Moderna) were messenger RNA (mRNA). Some vaccines (Janssen-Johnson & Johnson, AstraZeneca, Sputnik-V, and CanSino) were developed using human and primate adenovirus vectors. The vaccines from Bharat Biotech, Sinopharm, and Sinovac used the entire inactivated SARS-CoV-2 virus as technology.^1^

As expected, pregnant women were excluded from clinical trials for the development of vaccines against COVID-19.^2^ However, the American College of Obstetricians and Gynecologists (ACOG) and the Society for Maternal-Fetal Medicine (SMFM) recommended that all pregnant, postpartum, and breastfeeding women be vaccinated against the SARS-CoV-2 virus.^3^

Some studies have found that vaccination against COVID-19 induces an effective neutralizing antibody titer response in blood and breast milk.^4,5^ Romero Ramírez et al.,^5^ in a prospective cohort study with a convenience sample of 122 participants (98 vaccinated during lactation and 24 controls), observed that all vaccinated lactating women presented with anti-SARS-CoV-2 antibodies in serum and milk samples. Guida et al.,^4^ in a prospective cohort study, collected milk and serum samples from ten lactating women immunized with the mRNABNT162b2 vaccine. The authors detected anti-SARS-CoV-2 antibodies 20 days after application of the first dose of the vaccine in all serum samples; however, only in two milk samples, they detected a low level of antibodies. They also found the presence of anti-SARS-CoV-2 antibodies in all serum and milk samples seven days after the application of the second dose of the vaccine.

Nevertheless, it is crucial to emphasize that studies that reviewed the presence of antibodies against the new coronavirus in breast milk after vaccination are scarce, especially in Brazil. Furthermore, most of the papers reviewed the quantity of antibodies in women who had recovered from COVID-19^6,7^ and who had not been vaccinated and/or analyzed the breast milk and/ or the blood alone.^8-10^

Given this, there was a need to elucidate the expression of antibodies in the milk and blood of breastfeeding women vaccinated against the new coronavirus. Therefore, this study aimed to compare the presence of neutralizing antibodies against SARS-CoV-2 found in the breast milk and blood of vaccinated lactating women with those women who were not vaccinated.

## METHOD

**Figure 1:**
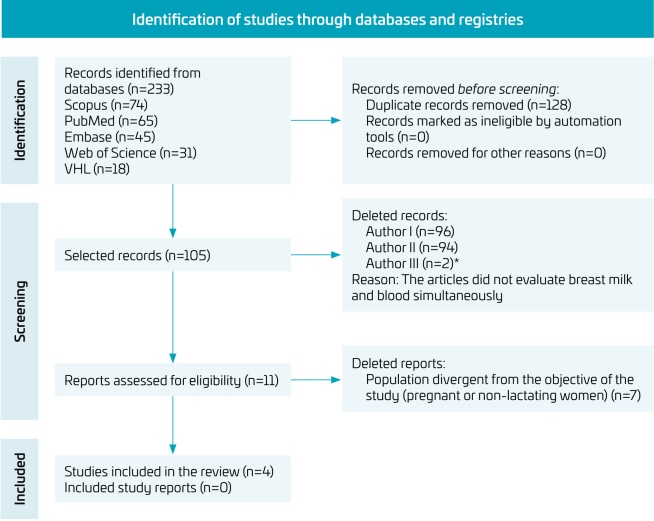
Flowchart of the article selection process.^11^

A systematic review was undertaken and registered in the Prospective Register of Systematic Reviews (PROSPERO) under CRD42021287554. The study was conducted in accordance with the Preferred Reporting Items for Systematic Reviews and Meta-Analyses (PRISMA) guidelines^11^ detailed in Figure 1.

*Assessment of conflicts.

Source: Page et al.^11^

From the definition of the key question, a systematized search strategy was developed using the Health Sciences Descriptors (DeCS) in the information sources available in the Virtual Health Library (VHL), the Medical Subject Headings (MeSH) indexing articles in Medical Literature Analysis and Retrieval System Online (Medline/Pubmed), and Emtree descriptors for searching in Embase. For the multidisciplinary databases Web of Science and Scopus, the strategy wording was adapted.

Database searches were carried out in July 2023 by two independent researchers in order to identify all studies that contained the desired keywords, including all combinations and free terms of the words: COVID-19, human milk, vaccine, and antibodies, and their Portuguese counterparts. The descriptors were combined with each other through the Boolean connector OR, within each set of terms, and then crossed with the Boolean connector AND. No restrictions on publication period and language were set. The search strategy is available with the corresponding author.

Observational cohort, case-control, or cross-sectional studies that assessed the presence of neutralizing antibodies against SARS-CoV-2 in the milk and blood of vaccinated breastfeeding women as well as unvaccinated breastfeeding women (control group) were qualified as eligible. Case reports, systematic reviews with or without meta-analysis, articles that analyzed antibodies in infected or non-lactating women, and studies that did not provide comparison outcome data between groups that received or did not receive the vaccine were excluded.

The search strategy outlined above was used to obtain titles, abstracts and full texts of studies likely to be relevant for the review. Two reviewers manually and independently screened against predefined inclusion criteria to identify studies regarding the presence of neutralizing antibodies in the milk and blood of nursing mothers vaccinated against COVID-19. Review conflicts were settled by a third reviewer.

The risk of bias among the articles included was also determined independently by the same reviewers based on the Newcastle-Ottawa Scale (2021) assessment tool. This scale contains eight items in three domains: group selection (0–4 points), goodness of fit for confounding (0–2 points), and assessment of exposure after outcome (0–3 points). The score for this tool varies from 7 to 9, 4 to 6, and 0 to 3, being considered high, moderate, and low quality, respectively.^12^

Data from the selected articles were tabulated according to the following items: name of authors, year, country, study design, vaccine technology and commercial name, follow-up time, sample size of vaccinated breastfeeding women, sample size of unvaccinated breastfeeding women, eligibility criteria, exclusion criteria, types of antibodies found in the blood and milk of the vaccinated and unvaccinated group, limitations, and main results.

Due to the heterogeneity between studies, a meta-analysis of the papers included in our survey was not feasible. However, the results were systematically summarized and reported.

## RESULTS

Database searches identified 233 records, of which 128 duplicates were removed. Titles and abstracts of the remaining 105 papers were read and 94 were excluded. After reading 11 articles in full, four studies were considered eligible and, therefore, included in this systematic review. The study selection process was described in the PRISMA flowchart (Figure 1).

All selected papers were cohort studies and had a follow-up period of postpartum women between 43 and 121 days. The four studies included were conducted in Spain, Israel, the United States of America, and Poland, and were published in 2021. Details of the studies and population characteristics are presented in Table 1.

**Table 1:** Characteristics of selected studies on the effect of vaccines on the presence of neutralizing antibodies found in the milk and blood of lactating women immunized against SARS-COV-2, 2023.

Reference/country of study	Study design	Vaccine technology (trade name)	Follow up time (days)	Sample size		Eligibility criteria	Exclusion criteria

				Vaccinated lactating women	Unvaccinated lactating women		
Jakuszko et al.,^13^ Poland	Cohort	mRNA (Pfizer)	43	32	28	Vaccinated and unvaccinated lactating women against COVID-19	Lactating women previously infected with SARS-CoV-2
Nir et al.,^15^ Israel	Cohort	mRNA (Pfizer)	59	64	11	Lactating women aged ≥18 years who received two doses of the BNT162 b2 mRNA vaccine against COVID-19 at least 14 days before childbirth	NI
Romero Ramírez et al.,^5^ Spain	Cohort	mRNA (Pfizer and Moderna)	62	100	24	Lactating healthcare workers who were breastfeeding their child at the time of SARS-CoV-2 vaccination	Participants with HIV infection, illnesses or treatment that cause immunosuppression, previous infections or ongoing symptoms compatible with COVID-19 at the time of recruitment
Valcarce et al.,^14^ United States	Cohort	mRNA (Pfizer and Moderna)	121	22*	22	Lactating women with no known history of COVID-19 infection; ≥18 years old, able to provide informed consent and receiving COVID-19 vaccine during lactation	Lactating women who did not complete sample collection

mRNA: messenger RNA; HIV: human immunodeficiency virus; NI: not informed.

*The group of vaccinated lactating women is the same group of unvaccinated (i.e., unexposed) women; however, before immunization.

There was variability in the sample size of the selected studies ranging between 22 and 124 women. The mean/median maternal age was greater than 30 years. All breastfeeding women received two doses of immunization against the SARS-CoV-2 virus using mRNA technology vaccines (Pfizer-BioNTech or Moderna), as recommended by each manufacturer. In none of the studies, vaccine booster doses were given to lactating women.

The evaluation of the quantity and type of antibodies found in the milk and blood of lactating women after vaccination was carried out differently in all four studies. Jakuszko et al.^13^ performed the evaluation four times, Valcarce et al.^14^ twice, while Nir et al.^15^ and Romero Ramírez et al.^5^ just once. The antibodies evaluated and the main outcomes are presented in Table 2.

**Table 2:** Characteristics of technologies used, antibodies evaluated, and main results.

Reference	Vaccinated lactating women		Unvaccinated lactating women		Main results

	Antibodies in the blood	Antibodies in milk	Antibodies in the blood	Antibodies in milk	
Jakuszko et al.^13^	IgM=ND	IgM=ND	IgM=ND	IgM=ND	The response to vaccination against SARS-CoV-2 is higher after the second dose. The levels of IgA and IgG antibodies in breast milk and serum of mothers after the COVID-19 vaccine were positively correlated. None of the serum and milk samples was positive for anti-SARS-CoV-2 IgM antibodies.
	IgG Day 8±1=4.62±3.57 Day 43±4=3,276.5±1,803.3	IgG Day 8±1=ND Day 43±4=6.43±4.33	IgG Day 8±1=3.41±0.80	IgG Day 8±1=ND	
	IgA Day 8±1=0.33±0.37 Day 43±4=9.37±8.08	IgA Day 8±1=0.55±0.32 Day 43±4=2.73±2.13	IgA Day 8±1=0.17±0.07	IgA Day 8±1=0.69±0.39	

Nir et al.^15^	IgM=NI	IgM=NI	IgM=NI	IgM=NI	All blood and breast milk samples were positive for anti-SARS-CoV-2 IgG. Likewise, 98% of umbilical cord blood samples and 96% of neonatal blood were positive for anti-SARS-CoV-2 IgG.
	IgG=26.1 (22.0–39.7)	IgG=4.9 (3.8–6.0)	IgG=2.6 (0.9–3.5)	IgG=ND	
	IgA=NI	IgA=NI	IgA=NI	IgA=NI	

Romero Ramírez et al.^5^	IgM=NI	IgM=0.04±0.08	IgM=NI	IgM=0.01±0.00	All vaccinated mothers developed anti-SARS-CoV-2 IgG antibodies in serum and milk samples. All breast milk samples contained IgG antibodies and 89% of them contained IgA antibodies. Highly significant correlation between antibody levels in serum and breast milk samples.
	IgG=3,379.64±1,639.46	IgG=12.19±11.74	IgG=0.41±0.37	IgG=0.02±0.05	
	IgA=NI	IgA=1.73±1.59	IgA=NI	IgA=0.21±0.08	

Valcarce et al.^14^	IgM=NI	IgM=NI	IgM=NI	IgM=NI	Vaccines against COVID-19 induce the secretion of IgA and IgG in human milk. The peak of anti-SARS-CoV-2 IgA and IgG in human milk and plasma occurred seven to ten days after the second dose of the COVID-19 vaccine.
	IgG After the 2^nd^ dose=4.90±0.13	IgG After the 2^nd^ dose=1.77±0.20	IgG=3.27±0.04	IgG=0.08±0.02	
	IgA After the 2^nd^ dose=3.66±0.05	IgA After the 2^nd^ dose=2.34±0.18	IgA=3.23±0.01	IgA=1.41±0.12	

ND: not detected; NI: not informed; IgM: immunoglobulin M; IgA: immunoglobulin A; IgG: immunoglobulin G.

Based on the immune response to vaccination against the SARS-CoV-2 virus among vaccinated compared to unvaccinated breastfeeding women, it was found that after vaccination with the Pfizer-BioNTech or Moderna immunizers, all blood and breast milk samples from the immunized women were positive for IgG antibodies. It was also observed that IgG levels were higher in the blood than in breast milk.^5,13-15^

It was also found that vaccinated breastfeeding women had higher levels of IgA in serum and milk than those who did not receive immunization and had an increase in IgA results after a second dose.^13,14^

Only two studies analyzed immunoglobulin M (IgM) in samples. Jakuszko et al.^13^ found that this antibody was undetectable in all samples. Romero Ramírez et al.^5^ evaluated IgM only in breast milk and observed an increase in its quantity among vaccinated breastfeeding women.

All selected articles had a score of eight points indicating high methodological quality performance (Table 3).

**Table 3:** Newcastle-Ottawa Scale quality assessment of each cohort study included.^12^

	Jakuszko et al.^13^	Nir et al.^15^	Romero Ramírez et al.^5^	Valcarce et al.^14^
Selection				
Representativeness of exposed cohort	0	0	0	0
Unexposed cohort selection	1	1	1	1
Determination of exposure	1	1	1	1
Demonstration that the outcome of interest was not present at the beginning of the study	1	1	1	1
Comparability				
Adjustments for the most important risk factors	1	1	1	1
Adjustments for other risk factors	1	1	1	1
Outcome				
Determination of outcome	1	1	1	1
Was the segment sufficient for the outcome to occur?	1	1	1	1
Adequacy of cohort monitoring	1	1	1	1
Total quality score	8	8	8	8

## DISCUSSION

Until the registration date in PROSPERO, this is the first systematic review that compared the antibodies found in the blood and human milk of breastfeeding women vaccinated against the SARS-CoV-2 virus with those not vaccinated.

Vaccination works as the entry of non-pathogenic antigens in the blood that interact with the immune system in charge of the body’s defense^16^ and causes the production of antibodies which are glycoprotein molecules capable of detecting, recognizing, and eliminating the antigen.^17^ Antibodies have five basic forms called immunoglobulins (IgG, IgA, IgM, IgD, and IgE)^18^ that can be found in various body fluids such as saliva, gastrointestinal fluid, blood, or breast milk.^19^

Breast milk offered to newborns is extremely rich in both nutritional composition and non-nutritive bioactive factors, such as antibodies that contribute to reducing infant morbidity and mortality through immunological protection as well as reducing contamination risk.^20,21^

The World Health Organization (WHO) recommends exclusive breastfeeding until six months of age and the continuation of breastfeeding combined with complementary foods for two years or more.^22^ Even considering the finding of SARS-CoV-2 viral particles in human milk, breastfeeding was recommended among women with suspected or confirmed disease given that viral transmission through human milk, if any, is quite rare^23^ and that its benefits for both women and newborns significantly outweigh the risk of transmitting this virus to newborns.^24^

Unanimously, the studies selected to compose this systematic review pointed out that after the use of mRNA vaccines produced by Pfizer-BioNTech and Moderna, lactating women presented different types of antibodies against the virus SARS-CoV-2 in blood and breast milk; they included IgG, IgA, and IgM antibodies.^5,13-15^ Furthermore, an increase in the number of antibodies was observed after the administration of a second dose of the vaccine, which confirms the importance of completing the vaccination schedule as recommended by the manufacturers.^13,14^

Regarding the types of immunoglobulins evaluated in the studies, it was observed that the IgM was not measured by Nir et al.^15^ and Valcarce et al.^14^. Jakuszko et al.^13^ did not detect this immunoglobulin in the milk and blood samples from lactating women (including those samples collected after vaccination). Only Romero Ramírez et al.^5^ detected IgM in breast milk samples, albeit in minimal quantities. This result was possibly due to IgM being found mainly when the infection is still active; it generally peaks in the first week after infection and disappears from the peripheral blood earlier than IgG.^25,26^

IgG was detected in the milk and blood of vaccinated lactating women who made up the sample of the four selected studies,^5,13-15^ while among those not vaccinated, IgG was minimally detected in two studies.^5,14^ It is interesting that IgG is generally the most abundant antibody in serum and is mainly synthesized in the secondary immune response to pathogens. IgG can activate the classical pathway of the complement system and is also highly protective.^27^

As for IgA, Jakuszko et al.^13^ and Valcarce et al.^14^ observed that vaccinated breastfeeding women had higher levels of IgA in serum and milk than unvaccinated women. Romero Ramírez et al.^5^ found that only human milk samples were positive for IgA in both groups. Nir et al.^15^ in turn, did not analyze the presence of IgA in any of the samples. IgA is the second most abundant immunoglobulin after IgG; it is present in external secretions such as saliva, tears, colostrum, and respiratory and intestinal secretions. Therefore, IgA is important in the primary immunological line of defense against local infections in sites such as the respiratory and gastrointestinal tracts.^27^

Efficient transfer of anti-SARS-CoV-2 IgG antibodies to newborns through the placenta of women vaccinated during pregnancy with BNT162b2 mRNA technology has been reported.^15^ Corroborating this result, Collier et al.,^28^ in a cohort study of pregnant women vaccinated against COVID-19 during pregnancy, also detected the presence of anti-SARS-CoV-2 IgG in maternal serum at birth and in the umbilical cord blood, suggesting the existence of efficient transplacental transfer of maternal antibodies.

Other vaccines, such as influenza, also increased antibodies in the milk and maternal blood of vaccinated women. Schlaudecker et al.,^29^ in a prospective study conducted in Bangladesh, analyzed the breast milk and serum of 340 women who received the influenza vaccine. The results of this study showed that IgA anti-influenza antibodies were higher in the breast milk and blood of mothers who received the vaccine. Furthermore, the sustained high levels of specific IgA in breast milk from women immunized during pregnancy suggest that breastfeeding may provide vaccine-specific local mucosal protection for the infant up to six months of age.

Abu Raya et al.,^30^ aiming to examine the levels of IgA and IgG specific to pertussis in breast milk, recruited 37 women (25 vaccinated with Tdap and 12 unvaccinated) to form a cohort. It was found that antibody levels against pertussis in breast milk were significantly higher among women vaccinated with Tdap during pregnancy than among those not vaccinated.

In this connection, based on the selected studies, we could verify that the vaccine against COVID-19, like other vaccines approved for use in pregnant and postpartum women, such as those against influenza, tetanus, diphtheria, and pertussis, increases the number of antibodies in the milk and blood of women who chose to receive immunization against the SARS-CoV-2 virus.^29,30^

In short, three studies included in this review^5,13,14^ found a highly significant correlation between the antibody levels in serum samples and breast milk. Thus, the presence of antibodies in the blood appears to predict the appearance of antibodies in human milk.

It is important to highlight that in order to confirm that mothers vaccinated against COVID-19 can transfer passive immunity to their newborns through breastfeeding, capable of potentially protecting them against SARS-CoV-2 infection, future studies are needed on the effects of vaccination on newborn antibodies and the neutralization capacity of these antibodies.

Another important aspect to be considered to elucidate the possible benefits of vaccination in maternal and child protection against the SARS-CoV-2 virus is what would be the ideal time to administer the vaccine (first, second, or third trimester of pregnancy, or even, during lactation). The study by Mithal et al.,^31^ carried out with women vaccinated against COVID-19 during pregnancy, demonstrated that the transfer of antibodies appears to increase with the latency of vaccination and suggested that, at least among women in the third trimester of pregnancy, early vaccination may produce greater neonatal immunity. However, Yang et al.,^32^ in a retrospective cohort study with 1,359 pregnant women, stated that immunization in any trimester of pregnancy, or before pregnancy, is associated with detectable levels of maternal anti-SARS-CoV-2 antibodies at delivery although the highest levels of maternal and consequently cord blood antibodies occur with vaccination performed early in the third trimester of pregnancy.

Since research on aspects of the SARS-CoV-2 virus is extremely important given the short period of discovery of the virus and simultaneously the importance of studying the effects of any and all immunizations, this systematic review provides an expanded view of the state of the art on the expression of maternal antibodies against COVID-19 in those vaccinated women, thus providing an assumption that vaccination is the most promising and safe way of maternal and child protection.

Regarding limitations, only two studies compared the number of antibodies between vaccinated and unvaccinated breastfeeding women; however, only Romero Ramírez et al.^5^ and Jakuszko et al.^13^ performed tests to gather evidence that those women were not infected or that they already had antibodies against COVID-19 in their blood at the time of collection. All studies reported the application of only mRNA-type vaccines; thus, we cannot generalize the results to other vaccine manufacturing technologies. Future studies should preferably evaluate the effect of other available vaccine technologies and the effect of booster doses of each of those vaccines. Valcarce et al.^14^ considered the unexposed group (unvaccinated breastfeeding women) as an exposed group at a later date, after the breastfeeding women took the vaccine against the new coronavirus. Nir et al.^15^ analyzed women who had recovered from COVID-19 with evidence of previous positive tests for SARS-CoV-2 as a control group. Furthermore, some significant differences in the studies included the participants’ sample size, the collection methods, and the variability in the number of times the samples were analyzed. Finally, all studies included in this review were international, thus confirming the scarcity of studies on this topic in Brazil.

In conclusion, it was found that after vaccination with the Pfizer-BioNTech or Moderna immunizers, all blood and breast milk samples from lactating women were positive for IgG antibodies, which were even more predominant in the blood. Likewise, vaccinated lactating women had higher levels of IgA in serum and milk than unvaccinated women. Furthermore, the immune response to vaccination against SARS-CoV-2 was improved after the administration of a second dose, that is, when breastfeeding women complete the vaccination schedule indicated by each vaccine manufacturer. Therefore, as breastfeeding women vaccinated against the SARS-CoV-2 virus showed higher antibody expression when compared with those not vaccinated, it is likely that, in addition to maternal protection against COVID-19, immunization also provides neonatal immunity through nursing. Thus, vaccination against COVID-19 in pregnant and postpartum women as well as nursing should be encouraged.
